# Focal Neurologic Deficit After Epidural Catheter Removal Leads to Meningioma Diagnosis

**DOI:** 10.7759/cureus.16015

**Published:** 2021-06-29

**Authors:** Morgan A Clond, Evin A Koleini, Timothy E Richardson, Stephanie A Zyck, Vandana Sharma, Mashaal Dhir, Fenghua Li, Satish Krishnamurthy, Sebastian Thomas, Xiuli Zhang

**Affiliations:** 1 Anesthesiology, State University of New York Upstate Medical University, Syracuse, USA; 2 Pathology, University of Texas Health Science Center at San Antonio, San Antonio, USA; 3 Neurosurgery, State University of New York Upstate Medical University, Syracuse, USA; 4 Anesthesiology and Pain, State University of New York Upstate Medical University, Syracuse, USA; 5 Surgery, State University of New York Upstate Medical University, Syracuse, USA

**Keywords:** epidural analgesia, focal neurologic deficits, elevated intracranial pressure, meningioma, central nervous system disorders, neuraxial block, postoperative problems

## Abstract

We present an unusual case of a 60-year-old female who developed subtle, new-onset left upper and lower extremity weakness on day five of perioperative thoracic epidural placement. The onset of a focal neurological deficit after epidural placement usually raises suspicion for the presence of an epidural hematoma, abscess, or traumatic cord lesion. However, in this patient, brain imaging revealed a large, previously undiagnosed intracranial mass. Classically, the risk of mass-related intracranial pressure shifts leading to neurological changes is associated with spinal techniques, including diagnostic lumbar puncture, combined spinal-epidural catheter analgesia, and unintended dural puncture during epidural placement. However, based on this case and our summary of case reports in the literature, we determined that symptom onset associated with an intracranial mass may also arise after apparently uncomplicated epidural placement. Symptom onset in our case series ranged from six hours to ten days and was highly variable depending on tumor location, with reported signs and symptoms including headache, vision changes, focal deficits, or alterations of consciousness. Further studies are required to establish definitive causation between the epidural technique and changes in cerebrospinal fluid pressures leading to symptom onset. Though rare, this is a time-sensitive diagnosis that must be considered for any patient with unexplained neurological findings after neuraxial anesthesia.

## Introduction

The differential diagnosis for focal neurological findings after neuraxial anesthesia includes epidural hematoma, epidural abscess, meningitis, traumatic cord lesion, and cauda equina lesion [1-3]. Epidural hematoma is the most common cause, with a variable rate of incidence depending on the characteristics of the population studied, ranging from 1/168,000 in obstetric epidurals [4] to 1/3,600 in elderly females undergoing knee arthroplasty [2]. Infectious and traumatic complications are comparatively less common but are prevalent enough to be reported in cross-sectional studies [1-3]. Intracranial processes such as stroke after cervical epidural [5] or after spinal anesthesia [6] or brain masses associated with neurological deficits after epidural or spinal anesthesia procedures [7-11] are rare and only reported as case reports.

Although the presence of a headache can sometimes help to guide the differential toward an upper versus lower motor neuron lesion, diagnosis is confounded in this situation by the relatively common occurrence of post-dural puncture headache. A high degree of vigilance and an open-minded approach to various differential diagnoses is warranted for patients with headaches after a neuraxial procedure, as intracranial masses can cause rapid decompensation. One case report describes the sequela of a labor epidural complicated by an unintended dural puncture. The initial complaints of headache were followed by fatal deterioration only two hours later. Imaging revealed an intracranial mass, but a tissue diagnosis was not obtained [12].

The contraindication to performing neuraxial anesthesia in the setting of elevated intracranial pressure (ICP), particularly in the presence of an intracranial mass, was initially based on case reports of herniation after diagnostic lumbar puncture procedures with large bore needles and large-volume cerebrospinal fluid (CSF) withdrawal. Indeed, the rate of herniation in the setting of bacterial meningitis is approximately 5% [13]. While it may be reasonable to infer a similar rate of risk for epidural or spinal anesthesia using modern equipment and techniques, the true risk remains unknown. In their review of the use of neuraxial techniques in the setting of intracranial hypertension or CSF shunting systems, Guerci et al. summarize 35 cases with intracranial hypertension of assorted etiologies undergoing spinal, combined spinal-epidural, or epidural. A total of 30 of these cases reported patient outcomes, which constituted six deaths, 22 uneventful outcomes, and two additional favorable outcomes after neurosurgical intervention. They posit that when a neuraxial technique is preferred for a given situation, it need not be dismissed out of hand, but rather that a decision should be made in consultation with neurology or neurosurgery based on the patient’s individual factors. Such factors include the size of the lesion and any edema or mass effect and whether the lesion is obstructive to the flow of CSF [14].

Epidural injections cause a transient increase in ICP, generally lasting three to seven minutes in healthy patients and producing no adverse effects [15]. However, in pathological states with elevated ICP, this increase can potentially compromise blood flow to the brain and spinal cord [16,17]. Hilt et al. measured the effect of extradural injection of local anesthetic on patients with altered cerebral compliance and demonstrated a significant increase in ICP due to lumbar dural sac compression with the cranial shift of CSF [17]. Thus, the shift of CSF may lead to changes in relative compartment pressures and thereby precipitate symptomatic presentation. Further studies would be necessary to establish this proposed mechanism as causal.

## Case presentation

A 60-year-old female patient with a past medical history of asthma and hyperlipidemia presented to our tertiary care hospital’s hepatobiliary surgery clinic with symptoms of nausea, vomiting, diarrhea, abdominal pain, and 10-pound weight loss. Magnetic resonance imaging (MRI) of the abdomen and pelvis demonstrated a 6.7 cm hypodense lesion of the pancreas which was concerning for possible neuroendocrine tumor. Biopsy samples collected under endoscopic ultrasound noted atypical cytology consistent with possible mucinous neoplasm. As this tumor type does not metastasize to the brain, but rather only to the chest, computed tomography (CT) of the chest was done and demonstrated no evidence of metastasis. Due to the interval increase in the size of the abdominal mass along with the patient’s progression of symptoms, the patient was scheduled for a pancreaticoduodenectomy (Whipple procedure).

For postoperative pain control, a thoracic epidural catheter was placed at the T9-10 interspace via a paramedian approach with loss of resistance to saline using a 17-gauge Tuohy needle. The needle placement was uncomplicated, and there was a loss of resistance at 6 cm with no return of CSF or blood. The catheter was threaded to a depth of 12 cm.

**Figure 1:**
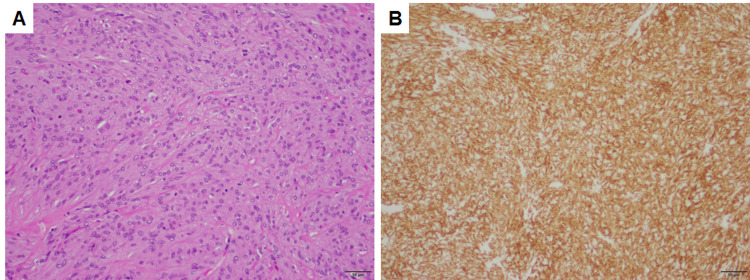
Pathology of the gastrointestinal stromal tumor. Gastrointestinal stromal tumor with (A) spindled and epithelioid cells with intervening collagen and (B) immunopositivity for DOG1. Both panels are taken at a total magnification of 100×, scale bars = 50 µm.

The operation proceeded uneventfully. Microscopic sections of the specimen demonstrated fascicles of spindled cells, some of which had epithelial features and background collagen (Figure 1A). The tumor cells were positive for DOG1 (Figure 1B) and CD117, consistent with gastrointestinal stromal tumor (GIST).

Postoperatively, an epidural infusion of 0.0625% bupivacaine with 2 µg/mL of fentanyl was initiated at a rate of 6 mL per hour. The epidural functioned well, providing analgesia without motor block. The next day, the patient was started on enoxaparin 40 mg daily for deep venous thrombosis prophylaxis. The patient was ambulating on postoperative day two. On postoperative day four, enoxaparin was held in anticipation of possible epidural removal. Platelet counts were within normal limits.

On postoperative day five, with the epidural infusion stopped, the patient noted subtle left upper and lower extremity weakness and complained of a mild headache. Nursing staff and physical therapists noted unequal gait phases on ambulation. Neurology was consulted and examination showed 4-/5 foot dorsiflexion, 4/5 foot plantarflexion, 4+/5 grip strength, and 4-/5 elbow extension on the left side. There were no other deficits on a complete neurological examination. The epidural was gently removed without difficulty and noted to be intact.

Unilateral deficit raises concern for either prolonged local anesthetic action or epidural hematoma. However, it was unlikely that either of these could be extensive enough to reach the upper extremity dermatomes but still only affect one side. Furthermore, the patient had no risk factors for epidural hematoma such as coagulopathy, renal dysfunction, or difficult epidural placement, and all guidelines were followed regarding the periprocedural timing of anticoagulant drugs. Due to the early time point and lack of any fever, leukocytosis, or tenderness at the epidural site, an epidural abscess was also thought to be unlikely. Thus, an upper motor neuron lesion was suspected. However, the patient had minimal risk factors for stroke, and no severe headache, mental status changes, aphasia, or cranial nerve deficits. The possibility of brain metastases was also considered but the incidence is extremely rare for GISTs.

Ultimately, a CT of the head without contrast demonstrated a 5.9 × 6.5 × 5.2 cm heterogeneously enhancing mass in the right frontoparietal lobe with a small amount of surrounding edema. There was a mass effect causing a midline shift of 2.5 cm at the level of the lesion, and mild effacement of the lateral and third ventricles. Neurosurgery was consulted and the patient was started on dexamethasone 4 mg every six hours to decrease edema around the tumor. Ten days after the initial Whipple procedure, a right parietal craniotomy was performed for intraventricular tumor resection.

**Figure 2:**
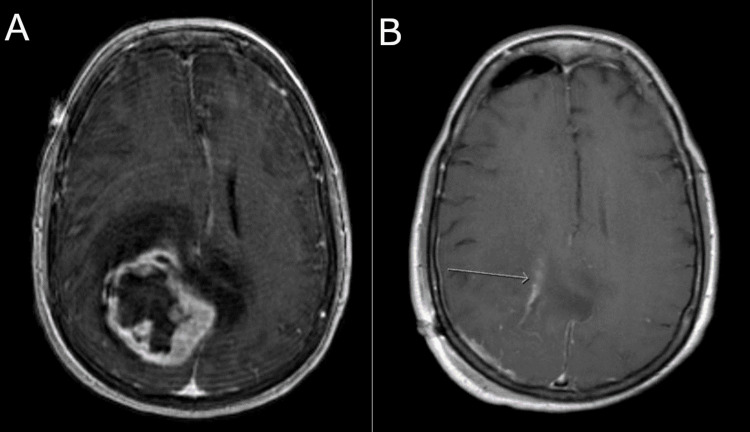
MRI of the brain. (A) Preoperative and (B) postoperative T1 MRI (axial view) with contrast. Postoperative imaging shows near gross total resection. MRI: magnetic resonance imaging

**Figure 3:**
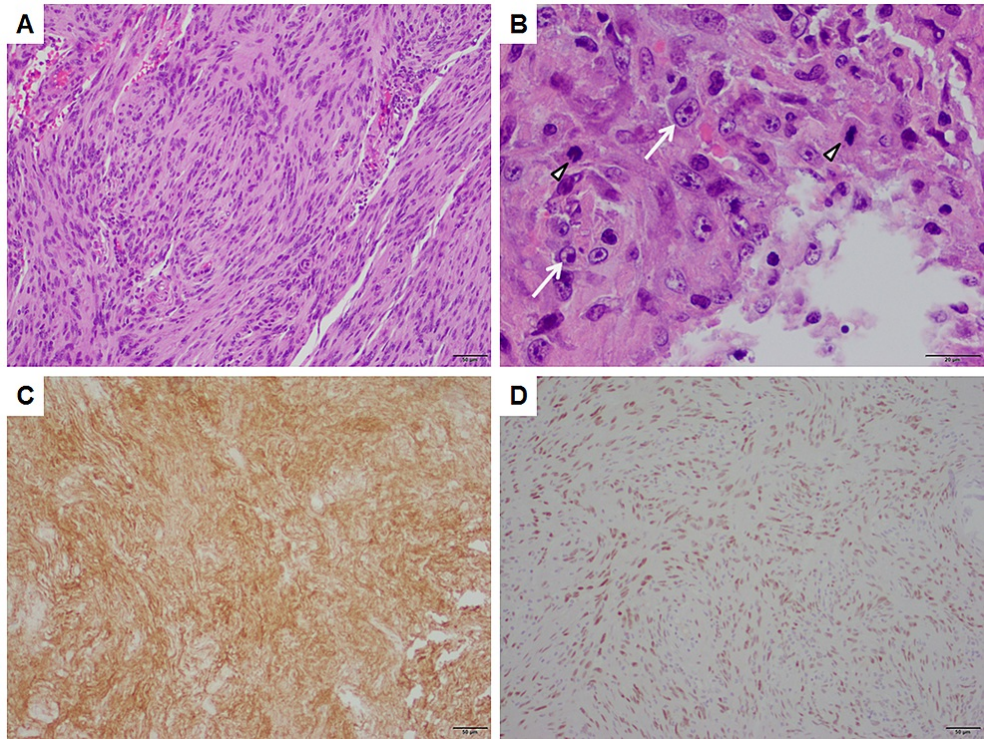
Pathology of the meningioma. Atypical meningioma, WHO grade II demonstrating (A) lobules and sheets of spindled and plump cells with (B) high-grade features, including elevated mitotic figures (arrowheads) and macronucleoli (arrows). The tumor is positive for (C) EMA and (D) PR, consistent with meningioma. Panels A, C, and D are taken at a total magnification of 100×, scale bars = 50 µm. Panel B is taken at a total magnification of 400×, scale bar = 20 µm. EMA: epithelial membrane antigen; PR: progesterone receptor

Postoperative MRI (Figure 2) demonstrated near gross total resection with a small amount of enhancement around the anterior aspect of the resection cavity. Microscopic sections demonstrated sheets and lobules of spindled to plump cells with syncytial cytoplasm (Figure 3A), background collagen, and scattered psammoma bodies, immunopositive for epithelial membrane antigen (Figure 3C), progesterone receptor (Figure 3D), and somatostatin receptor type 2a, consistent with a diagnosis of meningioma. Figure 3B demonstrates numerous WHO grade II features, including necrosis, loss of lobular architecture, prominent macronucleoli, increased mitotic figures (focally up to 6/10 HPF), and elevated Ki-67 proliferation index (focally greater than 30%), which is associated with a high rate of recurrence. Additional immunohistochemistry was negative for GIST tumor markers CD117 (c-KIT) and DOG1, demonstrating that her brain tumor was a second primary neoplasm and not metastasis of the GIST tumor. The patient was started on imatinib therapy under the care of the oncology service. Her neurologic examination improved postoperatively to the point of having no residual neurological deficits on follow-up.

## Discussion

This case report describes a rare incidental diagnosis after epidural analgesia. Compared with other reports summarized in Table 1, this is the first to describe this complication after thoracic epidural catheter placement and the first to report focal weakness as the presenting sign [7-11]. The previous case reports consist of four females and one male, ages 49 to 86, presenting for intra-abdominal or orthopedic procedures. Various neuraxial techniques are represented, including spinal, lumbar epidural, and combined spinal-epidural approaches. Interestingly, the onset of neurological symptoms was highly variable, from as few as six hours to up to ten days after surgery. All five cases presented initially with alterations of consciousness such as somnolence, agitation, disorientation, or behavioral changes. Progression to unresponsiveness occurred in three out of five cases. Pupillary changes were a late sign described in two cases. In the case where the meningioma was located in the sphenoid region, the patient presented with reduced optic acuity and optic atrophy, as well as hyponatremia and urinary incontinence, consistent with the possible syndrome of inappropriate antidiuretic hormone given the lesion’s position in deep brain structures. Cushing’s triad of hypertension, bradycardia, and Cheyne-Stokes respiration was observed in one case.

**Table 1:** A summary of case reports of symptomatic meningioma after neuraxial anesthesia. POD: postoperative day: CSE: combined spinal-epidural

Case	Technique	Onset, signs and symptoms	Tumor	Outcome	Source
64 F, Uteropexy	Spinal	POD 10, somnolence, fever, and meningeal signs due to intratumoral abscess	2.5 cm, frontal	Recovered to presurgical baseline	[7]
49 F, Knee replacement	Spinal	6 hours postoperatively, altered mental status, urinary incontinence, and hyponatremia	3.2 cm, sphenoid	Referred to neurosurgery	[8]
82 F, Hip surgery	L2-3 CSE	POD 2, dyspnea and delirium progressing to coma	5-6 cm, frontal	Death	[9]
86 F, Hip replacement	L3-4 Spinal	8 hours postoperatively, agitation and disorientation	Large, infratentorial	Complete recovery	[10]
55 M, Prostatectomy	L2-3 Epidural	POD 1, somnolence progressing to unresponsiveness Cushing’s triad	7 cm, frontal	Complete recovery	[11]
60 F, Whipple	T9-10 Epidural	POD 5, left-sided weakness	6.5 cm, parietal	Complete recovery	Current case

Although meningioma is the most common nonmalignant brain tumor, accounting for 37.6% of all central nervous system tumors [18], the probability of performing a neuraxial block in a patient with an undiagnosed meningioma is low. According to the US Central Brain Tumor Registry’s 2019 report, meningiomas have an average annual age-adjusted incidence rate of only 8.6 per 100,000 [18]. It is unknown what percentage of patients with undiagnosed meningioma receive uneventful neuraxial anesthesia. Longitudinal screening studies of the general population would require such a large sample size as to be prohibitive in both scope and cost. Furthermore, for patients with slowly growing masses, the presence of the mass per se may not represent an absolute contraindication to a neuraxial technique. Rather, it has been proposed that relative risk depends on the size and location of the mass with regard to whether it causes significant mass effect (high risk of herniation), moderate mass effect or obstruction of CSF flow (moderate risk), or no findings of increased ICP (minimal to no risk) [19].

Once recognized, various measures to reduce ICP may be required. Such measures may include elevation of the head of the bed, dexamethasone, mannitol, hypertonic saline, and optimized sedation [20]. Hypoventilation due to sedating medications such as opioids can lead to hypercarbia and vasoconstriction and should be avoided. Normocarbia is preferred, and hyperventilation to induce vasodilation should only be instituted as a temporizing last line therapy [21]. If noninvasive measures are inadequate, or there is mechanical obstruction of CSF flow, external ventricular drain or shunt placement may be required [14,20]. Based on patient and tumor characteristics, ultimately a craniotomy for tumor resection or radiation therapy may be warranted.

## Conclusions

The successful management of this complication ultimately hinges on an open-minded approach to the differential and early recognition, brain imaging, and neurology and neurosurgery consultation. The onset of neurological symptoms may occur after any form of neuraxial access, including intrathecal, inadvertent dural puncture, and bolused or continuous infusion thoracic or lumbar epidural catheters. Though intracranial pathology has long been considered an absolute contraindication to spinal anesthesia, it is time to broaden the consideration to epidural techniques as well. At present, the implementation of screening CT or MRI prior to neuraxial analgesia is likely cost-prohibitive, with a low risk-benefit ratio. However, there may be utility in performing ophthalmoscopy or ultrasound of the optic disc diameter. Primarily, there is no substitute for a thorough history and neurological examination before and following neuraxial procedures. Manifestations of an intracranial process may be quite difficult to distinguish from other possible postoperative or postprocedural complications. Alterations of consciousness in previously reported cases were often initially presumed to be due to polypharmacy or delirium, and attempts to treat with various pharmacologic reversal or sedating agents delayed brain imaging and diagnosis in three of the cases. Further investigations would be necessary to determine whether there is a causative relationship between the CSF pressure shifts of neuraxial access and the onset of signs and symptoms of an intracranial mass.
